# Short-Term Hurricane Impacts on a Neotropical Community of Marked Birds and Implications for Early-Stage Community Resilience

**DOI:** 10.1371/journal.pone.0015109

**Published:** 2010-11-30

**Authors:** Andrew B. Johnson, Kevin Winker

**Affiliations:** 1 University of Alaska Museum, Fairbanks, Alaska, United States of America; 2 Museum of Southwestern Biology, University of New Mexico, Albuquerque, New Mexico, United States of America; Dalhousie University, Canada

## Abstract

Populations in fragmented ecosystems risk extirpation through natural disasters, which must be endured rather than avoided. Managing communities for resilience is thus critical, but details are sketchy about the capacity for resilience and its associated properties in vertebrate communities. We studied short-term resilience in a community of individually marked birds, following this community through the catastrophic destruction of its forest habitat by Hurricane Iris in Belize, Central America. We sampled for 58 d immediately before the storm, 28 d beginning 11 d after Hurricane Iris, and for 69 d approximately one year later. Our data showed that the initial capacity for resilience was strong. Many banded individuals remained after the storm, although lower post-hurricane recapture rates revealed increased turnover among individuals. Changes occurred in community dynamics and in abundances among species and guilds. Survivors and immigrants both were critical components of resilience, but in a heterogeneous, species-specific manner. Delayed effects, including higher fat storage and increased species losses, were evident one year later.

## Introduction

Efforts to preserve biodiversity increasingly manage isolated ecosystem fragments set aside as reserves in a matrix of anthropogenically altered habitats. However, natural disasters are prominent worldwide, and most biological reserves will eventually incur a large natural disturbance [1]–[3]. Reserves must be designed and managed to sustain disasters, because such large-scale disturbances raise the probability of losing community members. It is increasingly recognized that ecosystems must be able to cope with disasters, and managing for resilience, rather than hoping to avoid natural disasters, is viewed as the most viable framework for managing both human and natural communities [3]–[6].

The resilience of a community—its ability to absorb change without substantial alteration or with tolerable levels of losses [4], [7]—depends on the intensity and size of the disturbance, persistence of populations in the original community, recruitment through immigration and reproduction, and attributes of potential colonists, including habitat preferences, dispersal ability [2], and timing of the disturbance with respect to reproduction [8]. Developing a resilience management framework requires an understanding of: 1) a community's potential for resilience; 2) the processes of resilience, such as the contributions of survivors and immigrants to the post-disturbance community; and 3) whether there are predictable aspects of a community's response to disturbance. Despite the important role that disasters play in natural communities, studies of their effects remain uncommon. This is due to the unpredictability of natural disturbances and to generally poor pre-disturbance baseline data. Among vertebrates, a management framework for avian populations impacted by hurricanes, especially small populations, has begun to emerge [9], [10], but an important gap remains in being able to track disaster-related phenomena at the level of the individual.

We had an unprecedented opportunity to study resilience in a community of individually marked, nonmigratory (resident) Neotropical birds in lowland forest in southern Toledo District, Belize, Central America. Our data from this natural trajectory experiment [11] demonstrate how a terrestrial vertebrate community responded to Hurricane Iris, and these data provide details of key parameters of resilience from the perspective of marked individuals that endured the disturbance. We show that the potential for resilience was initially high in this community; our data track resilience at the level of the individual; and our study may provide insight into some general responses of resilience.

## Methods

**Figure 1:**
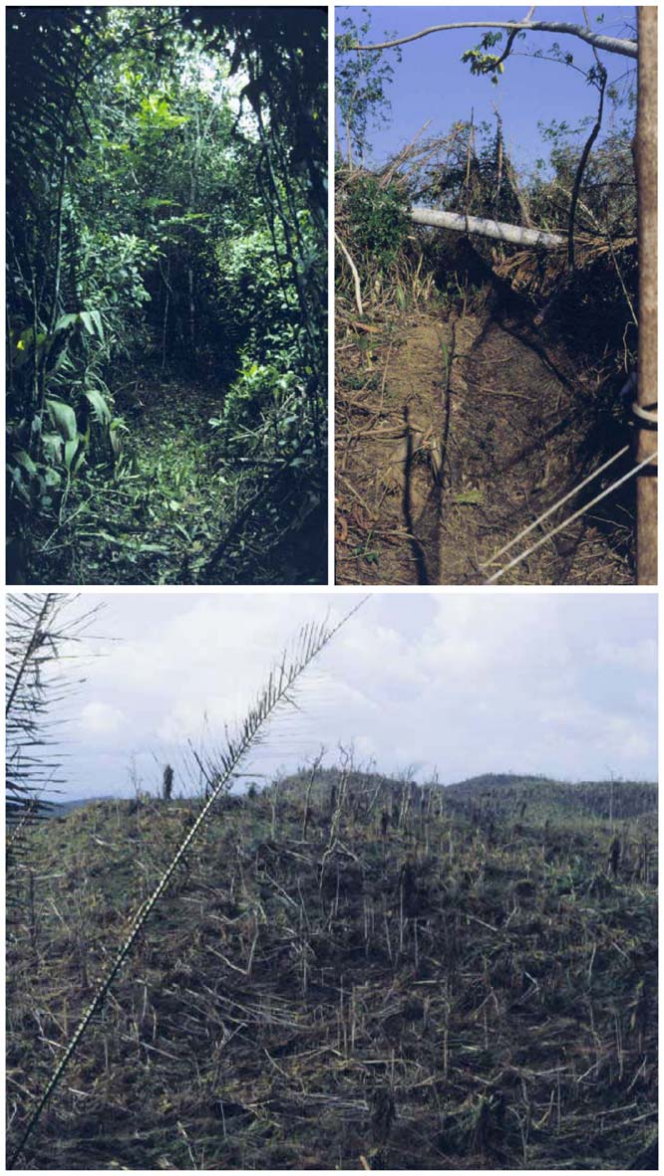
Habitat changes to lowland forest wrought by Hurricane Iris, which struck on 8 October 2001. Top left: A net lane during the Pre-Iris sampling period. Note shade and lush vegetation. Top right: A net lane during the Post-I sampling period. Note lack of shade and extensive damage to vegetation. Bottom: Typical damage to the lowland forest landscape caused by Hurricane Iris near Big Falls, Toledo District, Belize.

Our main study site (1.3 ha) was remnant primary forest joined with 25-year-old second-growth forest and edge. It was adjacent to a citrus orchard and was part of a matrix of human-influenced habitats in the floodplain of the Rio Grande near Big Falls, Toledo District, Belize (16° 15.8′ N, 88° 52.4′ W; elevation 20 m). We used mist nets to sample the understory bird community, capturing and banding birds for 58 days until the day before this site was heavily impacted by Hurricane Iris. We sampled the same site two more times following the hurricane. All sampling occurred during the wet season. Our first sampling period (Pre-Iris; 11 Aug - 7 Oct 2001; 8,805 net h) ended the day before Hurricane Iris, a Category 4 hurricane, ripped a 50 km-wide swath of destruction through southern Belize. With sustained winds of 230 km/h and gusts approaching 300 km/h, the storm caused massive destruction, leaving extensive areas of lowland forest a tangle of fallen trees (Figure 1). The effect on our site was to change the habitat from a nearly closed-canopy forest of 20 m to a 5 m-high tangle of uprooted and broken trees, branches, and vines (Figure 1). We next sampled 19 Oct - 15 Nov 2001 (Post-I; 1,114 net h), and, lastly, about one year later (Post-II: 8 Sep to 15 Nov 2002; 2,784 net h). One net h equals one 12-m mist net open for one h. Nets were set in two rows of 15 nets each, with rows and nets each spaced 30 m apart during Pre-Iris, alternating between 30 and 36 mm mesh size. During Post-I and Post-II, nets were placed on the site as closely as possible to the original net locations to adequately sample the entire Pre-Iris site, but placement was constrained by fallen trees. During Post-II we also sampled at an undisturbed site outside the hurricane zone. This secondary site was similar in forest age and structure to our main, pre-hurricane site and was located near Forest Home, Toledo District. We netted for 587 net h on this site during Post-II. Field work was conducted under appropriate permits (Belize Forest Department CD/72/2/01 & CD/60/2/02, and IACUC Protocol No. 00-33).

Guild membership and habitat preferences were based on field observations and standard references [12], [[13], Table S1]. We follow the nomenclature of the American Ornithologists' Union [14]. Nearctic-Neotropic migratory species were excluded because the study spanned the period of autumn migration at the site, though the hurricane likely affected the suitability of the site for these birds also [15]. We used 2×3 *G*-tests to examine changes in the percentages that different guilds contributed to community composition. We calculated species diversity for each sampling period using the Shannon Index of diversity [16], which takes into account the number of species present in each sample and the number of individuals of each species present in each sample. Changes in species diversity were examined using Bonferroni-adjusted pairwise *t*-tests [17]. To examine changes in capture rates among sampling periods, we calculated the variance of the capture rate of each sample period [18], then used this variance to conduct Bonferroni-corrected *t*-tests. Only morning captures (the only time of day sampled during all three sampling periods) from an equal number of days in each sampling period were used to control for capture biases due to time of day and number of days sampled. For these capture rate analyses, banded individuals from a prior sampling period recaptured during a later period were considered as 'new captures' the first time that they were recaptured.

To estimate the expected recapture rate in an undisturbed regime, we divided the Pre-Iris sample into two periods and compared the number of birds banded in the first half of the Pre-Iris sampling period that were recaptured in the second half of that period to the number of birds banded during Pre-Iris that were recaptured during Post-I. Breaking the Pre-Iris sample into two periods in this way served as an undisturbed control.

To examine year-to-year recapture rates, we compared the recapture rate of individuals banded during a preliminary study in 1999 that were recaptured during 2001 (Pre-Iris and Post-I combined) with birds banded in 2001 (Pre-Iris and Post-I) that were recaptured during Post-II. We limited recapture rate comparisons between 2001 and 2002 to include only the three species banded in 1999 and recaptured in 2001.

We define “local survivors” as individuals banded during the Pre-Iris sampling period and later recaptured during Post-I or Post-II. We estimated the “survivor composition” of the community during Post-I and Post-II as the percentage of captured individuals that were survivors. Survivor composition can be reduced by emigration or mortality of banded individuals, by immigration of unbanded individuals, and by juvenile recruitment (the latter during Post-II only). The post-hurricane recapture of birds banded during Pre-Iris and the survivor composition of the post-hurricane community measure different phenomena. Recaptures of survivors measures persistence, whereas survivor composition considers persistence in addition to levels of immigration and juvenile recruitment, reflecting the contributions of the Pre-Iris community to the Post-I and Post-II communities at the level of the individual.

To examine changes in fat scores, we used a Kruskal-Wallis test among the three sampling periods and Tukey-type nonparametric multiple comparisons [17]. Although the median fat score is the appropriate measure of central tendency for these ordinal data [19], [ but see 20], we present mean fat scores because all median scores of resident species were zero, and the means allow a better understanding of the changes observed.

## Results

We observed the following phenomena after Hurricane Iris: Extirpation of eleven formerly regularly-captured species; persistence of many marked individuals, their prevalence in the population varying by species (edge species showing highest persistence); an influx of open-habitat species; immigrants to the site; higher movement rates (fewer recaptures); and a community-wide increase in fat scores.

**Figure 2:**
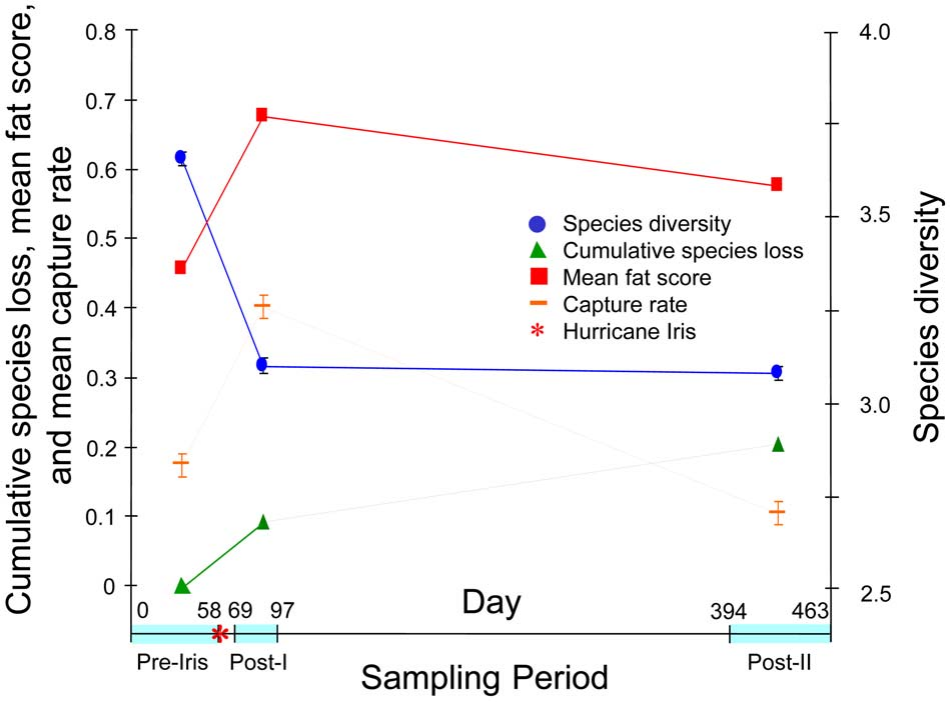
Changes in the avian community after Hurricane Iris. X-axis is number of days from beginning of study (11 August 2001), and sampling periods are highlighted (pale blue). A small proportion of species that were regular members of the Pre-Iris community were lost by Post-I, but these losses increased by Post-II (green). Species diversity (Shannon Index; blue) showed a significant decrease by Post-I and was still significantly lower during Post-II. Mean capture rate (captures per net h, an index of abundance) among non-granivore species (orange) increased significantly during Post-I, then decreased during Post-II to a level significantly below the Pre-Iris level. Mean fat score (red) among non-granivores increased significantly during Post-I and stayed above Pre-Iris levels during Post-II.

**Figure 3:**
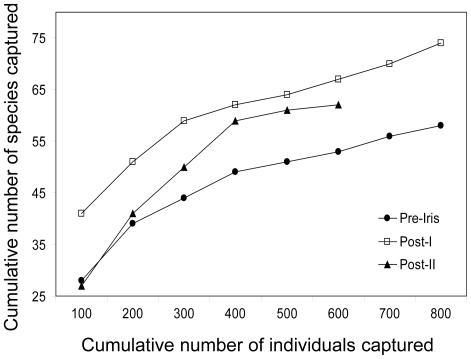
Species accumulation curves during the three sampling periods to contrast changes in community structure. The Post-I species accumulation curve had a much higher intercept than the Pre-Iris curve, but their shapes were the same. The Post-II curve was significantly different from both the Pre-Iris and Post-I curves, climbing more steeply and flattening more abruptly than the other two.

We captured over 2,000 individuals of 102 species, although many species were rare (<5 captures Pre-Iris). Many of the species captured prefer forest understory and comprise a group of conservation concern due to forest loss and fragmentation [[21], [22], Table S1]. Excluding rare species, we captured 53 species as regular members of the Pre-Iris community, and 44 of these were captured during all three sampling periods (Table S1). Just five (9%) of these 53 species were extirpated by Post-I, but this increased to 11 extirpated species (21%) by Post-II (Figure 2; Table S1). These species included forest understory specialists such as *Gymnocichla nudiceps*, *Onychorhynchus coronatus*, and *Henicorhina leucosticta* as well as species considered to prefer forest gaps, such as *Euphonia gouldi*, *Hylophilus ochraceiceps*, and *Arremon aurantiirostris* [[23], Table S1]. Ten of these 11 species were present at the forested site that we sampled outside of the hurricane zone during Post-II. Species diversity declined significantly between Pre-Iris and Post-I and remained significantly lower during Post-II (*t*-tests: *P*<0.001; Figure 2). The Post-I species accumulation curve had a much higher intercept than the Pre-Iris curve, but their shapes were the same (Kolmogorov-Smirnov test: *P*>0.1; Figure 3). The Post-II curve was significantly different from both the Pre-Iris and Post-I curves, climbing more steeply and flattening more abruptly than the other two (Kolmogorov-Smirnov tests: *P*<0.005; Figure 3).

**Figure 4:**
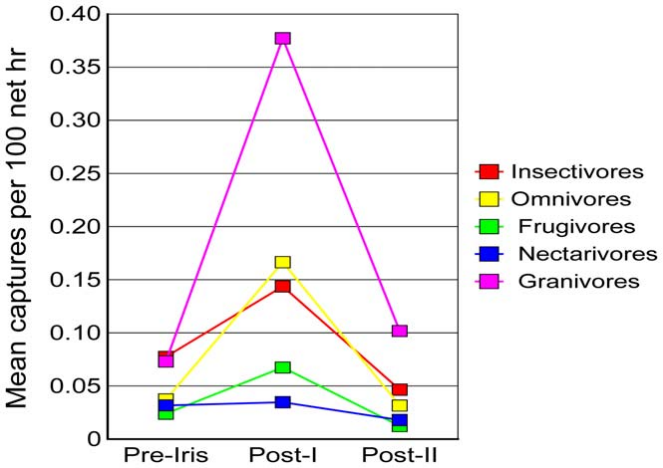
Guild-level changes due to Hurricane Iris. All guilds except Nectarivores showed a significant increase in Post-I. All guilds except Granivores had significant decreases to below Pre-Iris abundance levels by Post-II (see Table S2).

The capture rate of all species increased significantly during Post-I and declined to a level during Post-II that was not significantly different from the Pre-Iris rate (Figure 2). Mean capture rate among non-granivorous species increased significantly during Post-I, then decreased during Post-II to a level significantly below the Pre-Iris level (*t*-tests: *P*<0.05; Figure 2). Patterns of change among the different non-granivore guilds were generally similar to the pattern of all non-granivores combined (Table S2). The trend in capture rates among forest species and in all guilds except nectarivores and granivores was an increase from Pre-Iris levels during Post-I, and then a decrease to levels significantly below Pre-Iris during Post-II (Figure 4, Table S2). In nectarivores, the Post-I capture rate was not significantly different from the Pre-Iris rate, but the Post-II rate was significantly lower than the Pre-Iris and Post-I rates (Table S2, Figure 4). The granivore capture rate increased significantly Post-I, then decreased during Post-II, but to levels above the Pre-Iris rate (Table S2, Figure 4).

Gross habitat alteration caused mass immigration of open-habitat, granivorous species into the formerly forested site, and granivores increased from 21% of total captures during Pre-Iris to 46% and 53% of total captures during Post-I and Post-II, respectively (Table S2). New species were also captured during Post-I and Post-II. During Post-I, multiple captures of *Leptotila rufaxilla, Crotophaga sulcirostris, Ornithion semiflavum, Tityra semifasciata,* and *Chlorophanes spiza,* occurred; during Post-II, we recorded multiple captures of *Anthracothorax prevostii, Columbina talpacoti,* and *Todirostrum cinereum*—all species that had not been captured prior to the hurricane, although most were observed in the orchard and scrub near our study site.

Immigrants from beyond the study site (though not necessarily from beyond the zone of hurricane damage) were important in maintaining populations of some regularly occurring species (>5 captures Pre-Iris). Although there were no local survivors (recaptured banded birds) in 14 species during Post-I and in 26 species during Post-II, 70% of those species not represented by local survivors in Post-I or Post-II were still represented in the community by individuals not previously banded, demonstrating that immigrants were important to post-hurricane resilience (Table S1).

Survivors were also important to community resilience, and recapture during Post-I was a reasonable predictor of species persistence until Post-II. Of 32 Pre-Iris species with individuals recaptured during Post-I, 27 (79%) persisted to Post-II. Also, a species' presence in Post-I, whether through local (banded) or regional (unbanded) survivors, was a good predictor of presence during Post-II: 38 of 45 species (84%) were still present one year later (Table S1).

Post-hurricane recapture rates of banded individuals were lower than they had been before the hurricane. Comparison of the recapture rate of non-granivores within the Pre-Iris sampling period (dividing the sample into two periods) with their across-hurricane recapture rate (Pre-Iris to Post-I) showed a significant decline in recapture rate after Hurricane Iris (Table S1). Also, of 38 birds of 3 species banded during a preliminary study at the site in 1999, nine (24%) were recaptured during Pre-Iris. Of 370 individuals of the same species banded during Pre-Iris and Post-I, 18 (5%) were recaptured during Post-II. Recapture rates in these species were higher over 29 months before the hurricane than over 11 months afterwards, further evidence that Hurricane Iris caused lowered site fidelity.

Site fidelity and the contributions of local survivors to post-hurricane populations varied by species but were strongest among non-open-habitat species (Table S1). Of all non-granivore individuals banded during Pre-Iris, 18% were recaptured during Post-I and 5% during Post-II. The local survivor component of the post-hurricane community (the percentage of individuals that were banded during Pre-Iris and recaptured during Post-I or Post-II) was 25% during Post-I but had dropped to 14% by Post-II. Sixty-nine percent of species banded Pre-Iris were represented by local survivors in Post-I; this declined to 42% by Post-II (Table S1).

The species with the most recaptures and the highest local survivor components in post-hurricane populations were those often associated with edges or young second growth (i.e., disturbed, but not open, habitats); but many forest species showed values nearly as high, despite the lack of presumably suitable habitat on the site (Table S1). Among species with more than one individual banded during Pre-Iris, the following occurred: The highest Post-I recapture level of birds banded during Pre-Iris was 83% (*Thamnophilus doliatus*), and none of the ten species with the highest levels were open-habitat species (Table S1). During Post-II, the highest recapture level was 33% (*Tolmomyias sulfurescens*), and only one of the ten species recaptured most frequently favors open habitat (*Oryzoborus funereus*; Table S1). The highest survivor component in Post-I populations was 100% in two species (*Synallaxis erythrothorax* and *Saltator maximus*), and no open-habitat species occurred in the ten with the highest survivor components (Table S1). In Post-II the highest survivor component was 50% (*Synallaxis erythrothorax*), and the ten species with the highest survivor components included just one open-habitat species (*Oryzoborus funereus*; Table S1).

Community energetics also seemed to be affected by the hurricane. Fat levels among non-granivore species increased significantly between Pre-Iris and Post-I, then decreased between Post-I and Post-II, but to levels still above those found Pre-Iris (Tukey-type nonparametric multiple comparisons: *P*<0.05; Figure 2, Table S2). Because this change was consistent among all guilds but frugivores, it suggests that the difference was due to a change in community-level fat scores, and not an artifact of change in community composition (Table S2).

## Discussion

The severe habitat alteration that Hurricane Iris inflicted (Figure 1) caused short-term changes to the avian community. Species diversity dropped significantly during Post-I, and five regular members of the Pre-Iris community were lost (Figure 2). Most regularly-occurring species were still present during Post-I, illustrating a tremendous potential for long-term resilience in this avian community. Capture rates actually increased during Post-I (Figure 2), but an increased capture rate would be expected if the number of individuals on our site remained constant (i.e., direct mortality was low). The capture increase during Post-I could have been caused by a combination of a lowered canopy, making mist-net sampling more effective because it covered a greater vertical proportion of the post-hurricane habitat (supported by capture of canopy species not previously encountered, such as *Ornithion semiflavum* and *Chlorophanes spiza*; see also [9], [10]), increased foraging activity to maintain fitness in a changed environment (supported by an increase in fat scores), and an increase in territory size due to habitat degradation. This increase in capture rate suggests low direct storm-related mortality (see also [9]).

By Post-II, delayed effects clearly indicated a less diverse community comprised of reduced populations: cumulative species loss more than doubled to 11 regular members (Figure 2), the recapture rate remained low, the capture rate fell to levels significantly lower than prior to the hurricane (Figure 2), and the species accumulation curve changed significantly (Figure 3). Several species that occurred as regular or even common members of the pre-hurricane community were reduced to very low densities one year later (Table S1). These changes suggest that after one year the habitat was unsuitable to maintain populations of some forest-associated species that were present prior to the hurricane. It is likely that some species showing severe declines will be lost in the future, adding to cumulative species losses at this site until the forest recovers sufficiently for successful recolonization (Table S1; see also [24]). The presence of 10 of the 11 extirpated species at the non-damaged site indicated that these changes were due to Hurricane Iris and not to regional population fluctuations. Looking from the positive perspective, many forest species were able to persist at our main site after this major disturbance. The relatively high percentage able to do so one year after the storm (79%) may reflect at least in part a bird community adapted to the relatively high frequency of hurricanes in northern Middle America [25], [26]. Nevertheless, substantial negative short-term effects were evident, perhaps because strong hurricanes rarely strike this particular area (none recorded since at least the 1930s [27]), which enabled relatively old forest habitat to develop.

Hurricane Iris had a strong “stirring effect” on this bird community, and at multiple levels: community, species, and individual. The most striking example of this was the mass immigration of open-habitat granivores (e.g., *Oryzoborus, Sporophila,* and *Volatinia*). Capture rates in this guild tripled between Pre-Iris and Post-I, and granivores doubled as a percentage of the entire avian community during Post-I. This type of immigration into formerly forested habitats has been shown before in open-habitat granivores [25], [28] and in other species in the Caribbean [28], [29]. The lower recapture rate, the lowered capture rate of other community members, and the increasing cumulative species loss during Post-II also support a stirring effect, suggesting regional movements of former residents. Although regional population changes suggesting movement have been shown before [30], recaptures of marked individuals in our study showed that not all individuals left their original territories, and that a change to a nomadic existence did not occur for all individuals. Recapture rates decreased, though, suggesting that many of these birds new to our site continued moving, or that larger territories, which would reduce recapture probability [31], had been formed. Unaddressed on this time scale is whether for some species the site had become a sink—a habitat where reproductive replacement is not occurring, but instead populations remain only as a result of immigration.

A large percentage of the individuals on our study site were marked during Pre-Iris, which allowed us to examine the contribution of the local survivors to the post-Iris community. Many banded individuals were present after Hurricane Iris (1), and these “determined residents” played a strong role in populating the Post-I bird community. But immigrants and, by Post-II, juvenile recruits in some species also contributed to the recovering community.

Open-habitat granivores were seldom recaptured, suggesting a high degree of wandering after Hurricane Iris. However, most forest-associated species showed a mixed response to the hurricane, with a few individuals remaining on the site and others disappearing either through direct mortality (probably a minority) or through emigration. Those species with the highest degree of site fidelity were those associated with edge habitats (e.g., *Synallaxis erythrothorax, Thamnophilus doliatus*; Table S1), concordant with results from Nicaragua [32]. Many of the new species present during Post-I were canopy dwellers that may have been present during Pre-Iris but not effectively sampled by mist nets. Most of these species disappeared during Post-II, although *Leptotila rufaxilla*, which uses scrubby habitats, appeared to be an effective colonist. All of the new species captured during Post-II were species of early second growth and represented species that immigrated to the new, disturbed habitat.

At the guild level, our evidence corroborated other hurricane impact studies [9], [10] in showing that frugivores and nectarivores had the most severe declines, showing just 51.4% and 56.6%, respectively, of the Pre-Iris capture rates by Post-II (Table S2). However, declines in insectivores were nearly as great, with capture rates of 60.3% of Pre-Iris rates one year later during Post-II (Table S2).

Changes in community energetics (Figure 2, Table S2), which probably reflect insurance fattening, or individual adaptation to less predictable foraging success [25], [33]–[35], were not expected to remain one year after the hurricane (Post-II). However, human disaster victims can also show lingering post-disaster effects [36]–[38].

The return of this bird community to its Pre-Iris state will depend largely on habitat recovery [see also 9], [ 39], [ 40], and the recolonization of lost species will depend on immigration, making distances to source populations in suitable habitat important. One of the challenges in managing for resilience is to maintain areas of suitable habitat large enough that a catastrophic event does not obliterate an entire reserve network and its source populations, although artificial immigration (restocking) has been recognized as a way to aid recovery of hurricane-damaged fish communities [41]. In our study, distances to undisturbed patches from which some immigrants could have originated were as little as about 20–30 km. However, given indications on and near our site (Figure 1) that habitat changes were suboptimal for many of our study species, together with evidence of considerable local (on site) survival, it is likely that many of the immigrants to this site were individuals displaced from other areas within the zone of hurricane damage. And, although the hurricane-damaged region around the study site appeared thoroughly blasted, on a microhabitat scale local topographic and habitat variation caused some heterogeneity in damage levels. Thus, including consideration of substantial on-site survival (where damage was high), it is likely that immigrants emerged not from a simple “damaged/undamaged” landscape, but rather from a complex “damage mosaic” landscape that merged at some distance (ca. 20–30 km) into undamaged habitats.

Habitat mosaics and reserves designed to include multiple stages of successional forest recovery have been an important focus of wildlife management in hurricane-prone regions [9], [10]. An important insight provided by our data is that for some species even heavily damaged habitats retain potential as a post-catastrophe survivor and immigrant source. By demonstrating substantial local, individual survival in a high-damage area with marked individuals, our study shows that in many species the heavily damaged region itself can be an important population source for short-term, post-disaster recovery before any reproduction occurs. The distinction between source populations and temporary refugia becomes important through time and is sometimes overlooked [42]. Undisturbed habitat is already likely to be at carrying capacity, and, as our study shows, heavily disturbed habitat, although initially important, might have rapidly decreasing suitability as a refugium in some species. A habitat mosaic that blends heterogeneously from full- to zero-impact is thus a useful framework in which to consider how storm-related management might be scaled from individual to population levels.

By obtaining the first details of how marked individuals in a natural community of vertebrates respond to catastrophic disturbance, our study reveals the contributions of local survivors and immigrants to the post-disturbance community. When our data from marked individuals are combined with other natural disaster studies, a better understanding of resilience in vertebrate communities emerges (Table 1), augmenting reviews such as [9] and enabling improved predictions of the effects of catastrophic disturbance. This perspective (Table 1) also provides an evidence-based framework [43] within which to work toward disaster mitigation goals. For example, the large number of survivors in our study shows that the initial capacity for resilience is much higher than a visual assessment of habitat change would suggest, but that this capacity diminishes within one year. Our study also allows an understanding of how the recovering community and its component species coalesce from a combination of prior residents and immigrants.

**Table 1:** Some responses of vertebrate communities to catastrophic disturbances revealed by this and other studies.

Response	Basis
1) Community dynamics are altered: species diversity and species accumulation curves change.	[41] on fishes; Figures 2 & 3.
2) Species are lost, but at a smaller magnitude than the degree of habitat alteration.	Species losses in birds [28], [29], [32], [44]; magnitude small compared to habitat change: this study.
3) Abundances fluctuate at the community level and among species and species groups. Carrying capacity is lowered for some species. Some species preferring changed habitat become abundant.	Abundance changes in birds, frogs, lizards, and mammals [25], [ 27], [ 45], [[46], this study, Table S1; Figure 4]; lowered carrying capacity in birds [[47], this study, Table S2; Figures 2, 3]; increase of some bird species [[28], [29], [ 47]., this study, Table S1]
4) “Determined residents”: Strong individual site fidelity occurs through storm and continues long afterwards despite drastic habitat changes.	In humans [48] and birds (thisstudy, Table S1).
5) “Stirring effect” occurs among individuals: Individual mobility increases at the community scale.	Changed recapture rates (this study);suggested at population level by regional post-disaster shift in habitat use in lizards and birds [25], [30], [44], [47], [49].
6) Both survivors and immigrants comprise components of resilience in post-disaster populations, but in a heterogeneous, species- specific manner. Immigrants include new species.	This study; new species as colonists in birds [[29], [47], this study].
7) Heavily damaged habitat can provide survivors and immigrants.	This study (Table S1).
8) Delayed effects occur: Recolonization takes time, and delayed species losses occur.	In birds and lizards [[28], [39], [46], this study, Figure 2].
9) Energetic regime shift can occur: Individual fat storage increases and remains higher one year later.	Fat storage increase in birds [[25], this study, Figure 2].
10) Some formerly common or regular community members now present in very low densities.	This study (Table S1).

Whether management efforts can successfully work with these community responses to mitigate the effects of disaster is a different, though very important, question. For example, the delayed detrimental effects of habitat loss on local survivors provide an important window of opportunity for recovery. Capitalizing on this opportunity and effectively providing succor to these survivors could be an effective management approach when such actions are warranted, e.g., through a species' rarity or a reserve's isolation.

## Supporting Information

Table S1 **Tracking local survivors.** Fates of individuals of regularly occurring species banded Pre-Iris that were recaptured during subsequent sampling periods.
(PDF)

Table S2 **Guild-level changes in the avian community after Hurricane Iris.**
(PDF)
