# Supplementary material for: Short-Term Hurricane Impacts on a Neotropical Community of Marked Birds and Implications for Early-Stage Community Resilience
Source: PLoS One. 2010 Nov 30;5(11):e15109. doi: 10.1371/journal.pone.0015109 (PMC2994923; doi:10.1371/journal.pone.0015109)
Supplement: Table S1 — Tracking local survivors. Fates of individuals of regularly occurring species banded Pre-Iris that were recaptured during subsequent sampling periods. (PDF) [file pone.0015109.s001.pdf]

Table S1. Tracking local survivors: Fates of individuals of regularly occurring species banded Pre-Iris that were recaptured during subsequent sampling periods.

Species	Banded first half Pre-Iris	Recaptured second half Pre-Iris	Total banded Pre-Iris	Recaptured Post-I (%)[1]	Recaptured Post-II (%)[1]	Total captured Post-I	Post-I survivor composition (%)[2]	Total captured Post-II	Post-II survivor composition (%)[2]
All banded species*[3]	375	92	768	118 (15)	49 (6)	769	15	491	10
Non-granivorous species*	246	69	525	93 (18)	25 (5)	371	25	178	14
Insectivores	116	40	213	46 (22)	18 (8)	113	41	101	18
Omnivores	70	8	116	27 (23)	5 (4)	104	26	58	9
Frugivores*	75	24	227	28 (12)	5 (2)	145	19	28	18
Granivores*	112	19	206	16 (8)	21 (10)	384	4	294	7
Leptotila cassini[FF,4]	1	1	6	1 (17)	1 (17)	5	20	5	20
Phaethornis striigularis[FN,5]			7			0		0	
Phaethornis longiristris[FN,5]			30			2		0	
Amazilia tzacatl[FN,5]			43			9		23	
Amazilia candida[FN,5]			10			6		5	
Chloroceryle aenea[FP]	2	1	2	0 (0)	0 (0)	3	0	2	0
Pteroglossus torquatus[FO]	1	0	1	0 (0)	0 (0)	1	0	0	
Veniliornis fumigatus[FI]	5	3	6	3 (50)	1 (17)	4	75	5	20
Melanerpes aurifrons[SI]	0	0	1	0 (0)	0 (0)	0		0	
Synallaxis erythrothorax[SI]	12	3	19	2 (11)	2 (11)	2	100	4	50
Xenops minutus[FI]	1	0	3	1 (33)	0 (0)	3	33	0	

Table S1, continued.

Species	Banded first half Pre-Iris	Recaptured second half Pre-Iris	Total banded Pre-Iris	Recaptured Post-I (%)[1]	Recaptured Post-II (%)[1]	Total captured Post-I	Post-I survivor composition (%)[2]	Total captured Post-II	Post-II survivor composition (%)[2]
Dendrocincla anabatina[FI]	6	4	12	6 (50)	2 (17)	9	67	6	33
Dendrocincla homochroa[FI,5]			6			0		0	
Xiphorhynchus flavigaster[FI]	0	0	11	2 (18)	1 (0)	8	25	3	33
Taraba major[SI]	1	0	4	1 (25)	0 (0)	3	33	5	0
Thamnophilus doliatus[SI]	2	1	6	5 (83)	1 (17)	7	71	7	14
Cercomacra tyrannina[FI]	10	5	22	4 (18)	0 (0)	13	31	6	0
Gymnocichla nudiceps[FI]	5	4	12	3 (25)	0 (0)	4	75	0	
Formicarius analis[FI]	5	2	8	1 (13)	1 (13)	5	20	3	33
Myiopagis viridicata[FI]	6	1	7	3 (43)	0 (0)	7	43	9	0
Mionectes oleagineus[FF]	24	7	121	9 (7)	0 (0)	64	14	5	0
Poecilotriccus sylvia[FI]	4	1	9	1 (11)	1 (11)	2	50	5	20
Tolmomyias sulfurescens[FI]	2	1	6	2 (33)	2 (33)	12	17	6	33
Onychorhynchus coronatus[FI,5]			5			0		0	
Myiobius sulphureipygius[FI]	1	1	1	0 (0)	0 (0)	6	0	1	0
Myiozetetes similis[SO]			3	0 (0)	0 (0)	0		0	
Attila spadiceus[FI]			6	0 (0)	0 (0)	5	0	3	0
Pachyramphus polychopterus[FI]	3	1	8	0 (0)	1 (13)	1	0	4	25
Schiffornis turdina[FI,5]			5			0		0	
Manacus candei[FF]	50	16	93	18 (19)	4 (4)	49	37	17	24

Table S1, continued.

Species	Banded first half Pre-Iris	Recaptured second half Pre-Iris	Total banded Pre-Iris	Recaptured Post-I (%)[1]	Recaptured Post-II (%)[1]	Total captured Post-I	Post-I survivor composition (%)[2]	Total captured Post-II	Post-II survivor composition (%)[2]
Pipra mentalis[FF]	0		7	0 (0)	0 (0)	27	0	1	0
Hylophilus ochraceiceps[FI,5]			5			3		0	
Thryothorus maculipectus[FI]	17	3	25	7 (28)	4 (16)	11	64	14	29
Henicorhina leucosticta[FI]	6	2	6	0 (0)	0 (0)	0		0	
Ramphocaenus melanurus[FI]	9	2	10	3 (30)	2 (20)	7	43	12	17
Turdus grayi[FO]	10	0	26	0 (0)	0 (0)	29	0	10	0
Coereba flaveola[FN]			4	1 (25)	0 (0)	20	5	8	0
Eucometis penicillata[FI]	11	3	14	0 (0)	0 (0)	1	0	2	0
Habia fuscicauda[FO]	5	0	7	3 (43)	2 (29)	12	25	5	40
Ramphocelus sanguinolentus[FO]	1	0	1	1 (100)	0 (0)	3	33	4	0
Ramphocelus passerinii[FO]	23	4	30	15 (50)	0 (0)	41	37	15	0
Euphonia gouldi[FO]	1	0	4	1 (25)	0 (0)	5	20	0	
Volatinia jacarina[OG]	1	0	8	0 (0)	0 (0)	17	0	22	0
Sporophila americana[OG]	81	10	141	10 (7)	11 (8)	114	9	101	11
Sporophila torqueola[OG]	14	3	23	4 (17)	3 (13)	218	2	144	2
Oryzoborus funereus[OG]	16	6	34	2 (6)	7 (21)	35	6	27	26
Arremon aurantiirostris[FO]	4	1	6	2 (33)	0 (0)	3	67	0	
Arremonops chloronotus[FO]	10	1	13	1 (8)	2 (15)	3	33	8	25
Saltator coerulescens[SO]	2	0	4	1 (25)	0 (0)	2	50	3	0

Table S1, continued.

Species	Banded first half Pre-Iris	Recaptured second half Pre-Iris	Total banded Pre-Iris	Recaptured Post-I (%)[1]	Recaptured Post-II (%)[1]	Total captured Post-I	Post-I survivor composition (%)[2]	Total captured Post-II	Post-II survivor composition (%)[2]
Saltator maximus[FO]	11	1	15	3 (20)	1 (7)	3	100	10	10
Saltator atriceps[FO]	2	1	5	0 (0)	0 (0)	0		1	0
Cyanocompsa cyanoides[FO]	0	0	1	0 (0)	0 (0)	2	0	2	0
Amblycercus holosericeus[FI]	9	3	12	2 (17)	0 (0)	3	67	6	0

[1] Percentage of birds banded during Pre-Iris that were recaptured in sample.

[2] Percentage of birds banded during Pre-Iris present in sample.

[3] This total includes piscivores and nectarivores, and is not simply the sum of insectivores, frugivores, omnivores, and granivores.

[4] Habitat preference abbreviations: F: Forest; S: Scrub; O: Open-habitat. Guild abbreviations: I: Insectivore; F: Frugivore; O: Omnivore; P: Piscivore; N: Nectarivore; G: Granivore.

[5] Some or all individuals in some species, particularly hummingbirds, were not individually banded, so we were unable to examine site fidelity; numbers given are total captures in each period.
