# Supplementary material for: Short-Term Hurricane Impacts on a Neotropical Community of Marked Birds and Implications for Early-Stage Community Resilience
Source: PLoS One. 2010 Nov 30;5(11):e15109. doi: 10.1371/journal.pone.0015109 (PMC2994923; doi:10.1371/journal.pone.0015109)
Supplement: Table S2 — Guild-level changes in the avian community after Hurricane Iris. (PDF) [file pone.0015109.s002.pdf]

Table S2. Guild-level changes in the avian community after Hurricane Iris.

Comparisons of morning capture rates (captures ·neth^{-1}) during each sampling period[1]	*N*	Mean	SD	Community composition by guild during each sampling period	*N* (%)	Community composition of non-granivore captures	*N* (%)	Changes in fat scores	*N*	Mean fat score
All species								All species*		
Pre-Iris	373	0.2047[a2]	0.0348					Pre-Iris	610	0.192[a]
Post-I	712	0.7993[b]	0.0542					Post-I	653	0.326[b]
Post-II	338	0.2098[a]	0.0216					Post-II	261	0.463[c]
Non-granivore species				Non-granivore species*				Non-granivore species*		
Pre-Iris	292	0.1767[a]	0.0305	Pre-Iris	845 (79)			Pre-Iris	439	0.272[a]
Post-I	345	0.8424[b]	0.0469	Post-I	465 (54)			Post-I	276	0.678[b]
Post-II	144	0.1067[c]	0.0140	Post-II	272 (47)			Post-II	61	0.582b
Insectivores				Insectivores*		Insectivores		Insectivores*		
Pre-Iris	141	0.0774[a]	0.0140	Pre-Iris	334 (31)	Pre-Iris	334 (40)	Pre-Iris	162	0.086[a]
Post-I	128	0.1437[b]	0.0204	Post-I	156 (18)	Post-I	156 (34)	Post-I	75	0.187[a,b]
Post-II	75	0.0465[c]	0.0060	Post-II	112 (20)	Post-II	112 (41)	Post-II	20	0.25[b]
Omnivores				Omnivores		Omnivores*		Omnivores*		
Pre-Iris	68	0.0373[a]	0.0076	Pre-Iris	151 (14)	Pre-Iris	151 (33)	Pre-Iris	86	0.07[a]
Post-I	148	0.1662[b]	0.0198	Post-I	121 (14)	Post-I	121 (14)	Post-I	74	0.5[b]
Post-II	51	0.0316[c]	0.0056	Post-II	72 (13)	Post-II	72 (27)	Post-II	23	0.435[b]

Table S2, continued.

Comparisons of morning capture rates (captures ·neth⁻¹) during each sampling period[1]				Community composition by guild during each sampling period		Community composition of non-granivore captures		Changes in fat scores		
	N	Mean	SD		*N* (%)		*N* (%)		*N*	Mean fat score
Frugivores				Frugivores*		Frugivores*		Frugivores*		
Pre-Iris	44	0.0241[a]	0.0047	Pre-Iris	247 (23)	Pre-Iris	247 (29)	Pre-Iris	185	0.362[a]
Post-I	60	0.0674[b]	0.0139	Post-I	148 (17)	Post-I	148 (32)	Post-I	107	0.748[b]
Post-II	20	0.0124[c]	0.0032	Post-II	43 (8)	Post-II	43 (16)	Post-II	16	0.5[a,b]
Nectarivores				Nectarivores*		Nectarivores				
Pre-Iris	58	0.0318[a]	0.0069	Pre-Iris	105 (10)	Pre-Iris	105 (12)			
Post-I	31	0.0348[a]	0.0069	Post-I	39 (5)	Post-I	39 (8)			
Post-II	29	0.0180[b]	0.0052	Post-II	43 (8)	Post-II	43 (16)			
Granivores				Granivores*				Granivores*		
Pre-Iris	133	0.0730[a]	0.0169	Pre-Iris	219 (21)			Pre-Iris	175	0.16[a]
Post-I	336	0.3772[b]	0.0478	Post-I	400 (46)			Post-I	378	0.172[a]
Post-II	164	0.1018[c]	0.0100	Post-II	303 (53)			Post-II	200	0.485[b]

[1] Capture rates are from mornings only because it was the only time of day sampled during all three sampling periods.

[2] Sampling periods with the same letter are not significantly different from each other at a Bonferroni-adjusted $\alpha = 0.0167$.

* Denotes significant difference among sampling periods at $\alpha = 0.05$.
